# Comparative Performance of Oxygenation Indices for Mortality Prediction in Patients With Acute Respiratory Distress Syndrome at a Tertiary Care Intensive Care Unit in Bangladesh

**DOI:** 10.7759/cureus.116997

**Published:** 2026-09-27

**Authors:** Jannatun Naim, Nafiza Afroz, Most. Nasrin Sultana, Sabiha Binte Hossain, A. B. M. Borhan Uddin

**Affiliations:** 1 Department of Critical Care Medicine, Ministry of Health and Family Welfare, Dhaka, BGD; 2 Department of Anesthesia, Intensive Care, and Pain Medicine, National Institute of Burn and Plastic Surgery, Dhaka, BGD; 3 Department of Critical Care Medicine, Rajshahi Medical College Hospital, Rajshahi, BGD; 4 Department of Critical Care Medicine, Central Police Hospital, Dhaka, BGD; 5 Department of Critical Care Medicine, Bangladesh Medical University, Dhaka, BGD

**Keywords:** acute respiratory distress syndrome, mortality prediction, oxygenation index, oxygen saturation index, pf ratio

## Abstract

Background

Acute respiratory distress syndrome (ARDS) is a syndrome of acute inflammatory lung injury and impaired gas exchange, not hypoxemia alone, and carries high mortality. This study compared the discrimination of the partial pressure of arterial oxygen (PaO₂)/fraction of inspired oxygen (FiO₂) (PF) ratio, oxygenation index (OI), and oxygen saturation index (OSI), measured on the day of ARDS diagnosis, for intensive care unit (ICU) mortality in a Bangladeshi tertiary ICU.

Methods

This single-center prospective observational study enrolled 80 consecutive adults meeting the 2012 Berlin definition of ARDS and requiring invasive mechanical ventilation at Bangladesh Medical University between June 2024 and May 2025. Time zero (day 1) was the day the Berlin criteria were first met. Indices were calculated on days 1, 3, and 7 from simultaneous blood gas, oximetry, and ventilator data. Groups were compared by independent t- and chi-square tests, serial change by paired t-tests in survivors only, and discrimination by receiver operating characteristic (ROC) curve analysis with Youden cutoffs. Correlated areas under the curve (AUCs) were not formally compared, and no multivariable model was fitted.

Results

Forty-five of 80 patients (56.3%) died in the ICU, and 35 (43.8%) survived. Non-survivors had lower blood pressure, higher heart and respiratory rates, and greater ventilatory requirements (p<0.05). OI and OSI were higher and the PF ratio was lower among non-survivors (p<0.05). Discrimination was numerically highest for OSI (AUC=0.879; 95% CI: 0.786-0.977), followed by OI (AUC=0.867; 95% CI: 0.788-0.963) and the PF ratio (AUC=0.731; 95% CI: 0.612-0.881); as the intervals overlapped and no paired test was applied, this ordering is descriptive only. Specificity was 91.4% for both OSI and OI, and accuracy was 86.3% and 83.8% versus 70% for the PF ratio. Serial improvement among survivors was not statistically significant.

Conclusions

OI and OSI were higher among non-survivors and showed numerically higher discrimination for ICU mortality than the PF ratio, with OSI performing best. As the analysis was univariable and cutoffs were internally derived, these findings indicate discrimination and association rather than independent prognostic value, and the thresholds are exploratory. Larger multicenter studies with formal paired comparison of ROC curves, multivariable adjustment, and independent validation are warranted.

## Introduction

Acute respiratory distress syndrome (ARDS) is a syndrome of acute inflammatory lung injury with increased alveolar-capillary permeability and impaired gas exchange rather than hypoxemia alone [1-4]. The multinational LUNG-SAFE study reported that ARDS affects 10.4% of intensive care unit (ICU) admissions, with hospital mortality approaching 40% [1], and contemporary analyses indicate rates between 32% and 51% [2]. These figures are not directly comparable, because reported mortality depends substantially on the definition applied, the geographical and economic setting, case mix, the threshold for intubation, and the resources available for organ support. Comparisons across cohorts should therefore be read with that heterogeneity in mind.

Hypoxemia is nonetheless the component on which severity grading and outcome prediction rest, so accurate assessment of oxygenation remains central. The partial pressure of arterial oxygen (PaO₂)/fraction of inspired oxygen (FiO₂) (PF) ratio is the standard measure, with thresholds of 201-300 mmHg, 101-200 mmHg, and 100 mmHg or less defining mild, moderate, and severe ARDS [5]. It requires invasive arterial blood gas (ABG) sampling, and its value is influenced by the set FiO₂, level of positive end-expiratory pressure (PEEP), mean airway pressure, ventilatory mode, recruitment maneuvers, position, and sampling timing, so that identical lung injury can yield different values under different ventilatory conditions [6,7].

The oxygenation index (OI) incorporates mean airway pressure and so reflects both oxygenation and the support required [8]. Higher values are associated with mortality, longer ICU stay, and longer ventilation [9] and in COVID-19-related ARDS may outperform the PF ratio [10]. One caveat is important. Because mean airway pressure is clinician-set, the OI reflects the intensity of ventilatory treatment as well as intrinsic lung injury severity, so a high value may indicate an aggressive support strategy as much as a sicker lung. The OI also still requires ABG measurement.

The oxygen saturation index (OSI) replaces arterial oxygen tension with pulse oximetric saturation, making it non-invasive and continuously available [11]. It correlates strongly with the OI, is independently associated with hospital mortality [12], and has discriminated mortality better than PF ratio-based classification in adults [13]. Substituting peripheral oxygen saturation (SpO₂) for PaO₂ nevertheless carries costs. The oxyhemoglobin dissociation curve is sigmoidal, so saturations above roughly 97% correspond to a wide range of PaO₂, and the index loses resolution there. Pulse oximetry is further degraded by skin pigmentation, poor perfusion, vasopressors, motion artefact, anemia, and dyshemoglobinemias, with occult hypoxemia reported particularly in darker-skinned patients [10].

Comparisons among the three indices remain inconsistent, with some studies favoring the OI and others the OSI [8,13], and most available evidence is retrospective, drawn from high-income settings, or confined to subgroups such as COVID-19 ARDS. The gap is a specific one. To our knowledge, no prospective study has compared all three indices in the same unselected adult ARDS cohort, at a defined time zero, in a South Asian low- or middle-income ICU population. ICU mortality among ventilated patients in low- and middle-income countries ranges from 45% to 60%, well above high-income figures [14-16]. This excess mortality reflects several factors acting together. Patients frequently present late, the burden of sepsis is high, and intensive care beds, ventilators, trained staff, and organ support are all in short supply, while the case mix itself differs from that reported in wealthier health systems. Limited availability of ABG analysis also contributes, but it is one element among these rather than the principal cause. In Bangladesh, ICU capacity is concentrated in tertiary centers and mortality exceeds 50% [17], and the Kigali modification of the Berlin definition reflects the need for SpO₂-based alternatives [18]. The primary objective was accordingly to compare the discrimination of the PF ratio, OI, and OSI, measured on the day of ARDS diagnosis, for ICU mortality in a Bangladeshi tertiary ICU, with a secondary objective of describing their behavior over the first week.

## Materials and methods

Study design and setting

This was a single-center prospective observational study conducted in the ICU of the Department of Anesthesia, Analgesia, and Intensive Care Medicine at Bangladesh Medical University (BMU), Dhaka, Bangladesh. The study was carried out over a one-year period from June 2024 to May 2025. The ICU is a tertiary care referral center equipped with facilities for advanced respiratory support, including invasive mechanical ventilation and continuous monitoring of critically ill patients. Time zero for all analyses was defined as the day on which a patient first fulfilled the Berlin criteria for ARDS while receiving invasive mechanical ventilation in this ICU, and that day was designated day 1. Patients who already met the criteria at the moment of ICU admission had time zero at admission, whereas those who developed ARDS during the ICU stay had time zero at the point of diagnosis. Days 3 and 7 were counted from day 1 as defined above, not from the date of hospital admission. The outcome of interest was ICU mortality, defined as death occurring during the index ICU stay.

Study population

The study population comprised adult patients diagnosed with ARDS who were admitted to the ICU of the BMU during the study period. ARDS was defined according to the Berlin definition of 2012, and not according to the 2023 global definition of ARDS, because enrolment began before the global definition was adopted in this unit, which characterizes the syndrome by acute onset of respiratory failure within one week of a known clinical insult or worsening respiratory symptoms, the presence of bilateral pulmonary opacities on chest imaging not fully explained by pleural effusion, lobar collapse, or nodules, and respiratory failure not primarily attributable to cardiac causes or fluid overload [19]. In operational terms, the criteria applied at enrolment were onset within one week of a known insult or new or worsening respiratory symptoms, bilateral opacities on chest radiography or computed tomography not fully explained by effusion, collapse, or nodules, respiratory failure not fully explained by cardiac failure or fluid overload, and a PaO₂/FiO₂ ratio of 300 mmHg or less measured while the patient was receiving a PEEP of at least 5 cmH₂O. Severity was graded on the same PaO₂/FiO₂ measurement as mild (201-300 mmHg), moderate (101-200 mmHg), or severe (100 mmHg or less).

Inclusion and exclusion criteria

Patients aged 18 years or older with confirmed ARDS requiring invasive mechanical ventilation were eligible for inclusion. Both male and female patients were included, irrespective of the underlying etiology of ARDS. Patients with preexisting chronic respiratory diseases such as bronchial asthma or chronic obstructive pulmonary disease (COPD), as well as those with conditions that could interfere with accurate oxygen saturation measurement (including carbon monoxide poisoning or methemoglobinemia), were excluded from the study. All consecutive patients admitted to the ICU during the study period were screened against these criteria, and every eligible patient was considered for enrolment. No further selection was applied beyond the stated criteria and the availability of informed consent from the legal guardian or next of kin. The detailed inclusion and exclusion criteria applied for participant selection are summarized in Table 1.

**Table 1:** Inclusion and exclusion criteria applied for participant selection ARDS: acute respiratory distress syndrome; COPD: chronic obstructive pulmonary disease

Inclusion criteria	Exclusion criteria
Adult patients aged 18 years or older	Age below 18 years
Diagnosis of ARDS confirmed according to the Berlin definition	Preexisting chronic respiratory disease, such as bronchial asthma or COPD
Requirement for invasive mechanical ventilation	Conditions interfering with accurate oxygen saturation measurement, such as carbon monoxide poisoning or methemoglobinemia
Both male and female patients, irrespective of the underlying etiology of ARDS	Incomplete clinical, ventilatory, or arterial blood gas data required for the calculation of the oxygenation indices
Written informed consent obtained from the legal guardian or next of kin	Refusal of consent by the legal guardian or next of kin

Sampling technique and sample size

A consecutive sampling technique was used to recruit participants. Eligible patients were enrolled consecutively after applying predefined inclusion and exclusion criteria and providing written informed consent. The sample size was calculated via the standard formula for a single population proportion: $n = \frac{Z^{2} \times p \times q}{d^{2}}$. Using a 95% confidence level (Z=1.96), an expected proportion (p) of 0.24 for hospital mortality among ARDS patients, on the basis of a previous study by DesPrez et al., and a margin of error (d) of 0.10, the required sample size was calculated [11]. The complementary proportion (q) was taken as 1 − p = 0.76. Accordingly, the minimum required sample size was estimated to be 70 participants. Considering a 10% allowance for incomplete data, the final sample size was increased to 77 participants. The term dropout is deliberately avoided in this report because patients who did not contribute later measurements did so because they died or were discharged from the ICU, not because they withdrew from the study. However, a total of 80 patients fulfilling the eligibility criteria were enrolled during the study period. This calculation was designed to estimate a single proportion with acceptable precision. It was not designed to detect a prespecified difference between the areas under the receiver operating characteristic (ROC) curves of the three indices, no expected AUC difference was specified in advance, and no power calculation for a comparison of correlated ROC curves was performed before recruitment. The comparative findings reported below are therefore descriptive and exploratory, and the study should be regarded as adequately sized for the estimation of discrimination for each index individually but not as powered to establish a difference between them. We acknowledge that a calculation framed around a single proportion does not match a primary research question that is comparative rather than descriptive and that a calculation based on an expected difference between correlated areas under the curve (AUCs) would have been the appropriate design.

Data collection procedures

Data were collected prospectively via a predesigned, semistructured case record form. Baseline demographic information, including age, sex, and body mass index (BMI), was recorded at admission. Clinical parameters such as pulse rate, systolic and diastolic blood pressure, respiratory rate, and body temperature were recorded. Information regarding comorbidities, including diabetes mellitus, hypertension, ischemic heart disease, and chronic kidney disease, was also collected. The laboratory investigations included complete blood count and ABG analysis. Ventilator parameters, including tidal volume, PEEP, FiO₂, and mean airway pressure, were recorded. All patients were managed with volume-controlled mechanical ventilation via lung-protective strategies. The ventilation protocol was standardized across the unit. Ventilation was delivered in volume-controlled mode. The initial tidal volume was 6 mL/kg of ideal body weight, and the initial PEEP was 5 cmH₂O, raised in 2 cmH₂O increments as required while maintaining an inspiratory plateau pressure below 30 cmH₂O. FiO₂ was started at 100% and titrated downward to an oxygenation target of PaO₂ 55-80 mmHg or SpO₂ 88-95%. Where these targets were not met, the inspiratory-to-expiratory ratio was adjusted with monitoring for auto-PEEP and arterial carbon dioxide tension. If the plateau pressure exceeded 30 cmH₂O despite a tidal volume of 6 mL/kg, the tidal volume was reduced stepwise by 1 mL/kg to a minimum of 4 mL/kg of predicted body weight. Respiratory rate was adjusted to maintain adequate minute ventilation and an arterial pH between 7.30 and 7.45. All patients were deeply sedated or paralysed at the time mean airway pressure was measured, so that the recorded value reflected the set ventilatory support rather than spontaneous effort. Ventilator settings were not altered for the purpose of the study, and the ABG sample, the SpO₂ reading, and the ventilator parameters used to compute each set of indices were recorded simultaneously at the settings in use at that moment. Oxygenation indices were calculated as follows: the PF ratio, in mmHg, was calculated as PaO₂ (mmHg) divided by FiO₂; the OI was calculated as (FiO₂ × mean airway pressure in cmH₂O × 100) divided by PaO₂ in mmHg; and the OSI was calculated as (FiO₂ × mean airway pressure in cmH₂O × 100) divided by SpO₂ expressed as a percentage. In every computation, FiO₂ was entered as a decimal fraction, for example, 0.80 rather than 80, so that the multiplier of 100 returns values in the conventional clinical range. The OSI was computed from the SpO₂ value recorded at the time of measurement. Because the oxyhemoglobin dissociation curve is sigmoidal, SpO₂ values above approximately 97% correspond to a wide and indeterminate range of PaO₂, and the OSI is correspondingly less informative in that range. The mean day-1 SpO₂ was 77.58% in non-survivors and 91.53% in survivors, so the great majority of measurements lay well below the level at which this ceiling effect becomes material; the effect is nevertheless acknowledged in the limitations as an intrinsic constraint of the index. Data were recorded on days 1 (admission), 3, and 7 of the ICU stay.

Berlin definition and severity classification of ARDS

ARDS severity was classified according to the Berlin definition on the basis of the PF ratio measured under a minimum PEEP of 5 cmH₂O. Patients were categorized as having mild ARDS (PF ratio: 201-300 mmHg), moderate ARDS (PF ratio: 101-200 mmHg), or severe ARDS (PF ratio: ≤100 mmHg) [20]. The Berlin definition also defines ARDS as acute hypoxemic respiratory failure occurring within one week of a known clinical insult or worsening respiratory symptoms, with bilateral pulmonary opacities not fully explained by cardiac failure or fluid overload.

Handling of deaths and missing data

Because this point was not made explicit in the original description of the methods, it is set out here in full. Enrolment and day-1 data collection were complete. All 80 patients contributed a complete set of day-1 measurements of the PF ratio, OI, and OSI, and day 1 is the time point used for the primary comparison of the three indices, so no patient was excluded from that analysis, and no day-1 value was imputed. Incompleteness arises only at days 3 and 7, and it arises for a single reason. All three indices require a patient who is alive and still receiving invasive mechanical ventilation, since mean airway pressure is undefined once ventilatory support has been withdrawn. Forty-five of the 80 patients died during the observation period, and a further group had been successfully extubated and discharged from the ICU before day 7. Day-3 and day-7 data are therefore absent by clinical outcome and by design rather than through loss to follow-up, protocol deviation, or failure of data collection, and this is why a prospective study with complete enrolment nevertheless has incomplete later measurements. Serial analyses are confined to the 35 survivors for this reason. No imputation was performed for measurements that could not be obtained because a patient had died, since such values are undefined rather than missing. Missing values arising from any other cause, such as an arterial sample that could not be drawn at the scheduled time, were handled by analysis of available cases for the variable concerned, and the denominator is stated with each result.

Statistical analysis

IBM SPSS Statistics for Windows, Version 26.0 (IBM Corp., Armonk, New York, United States) was used for analysis. Continuous variables were tested for normality by the Shapiro-Wilk test and are described as mean±standard deviation, and qualitative variables are described as frequency and percentage. Survivors and non-survivors were compared by the independent samples t-test and by the chi-squared test, with Fisher's exact test applied where an expected cell count fell below 5. Test statistics are reported with their degrees of freedom and effect sizes as Cohen's d with a 95% CI or as Cramér's V. Age was categorized at 60 years for description only. Serial changes between day 1, day 3, and day 7 were assessed by paired t-tests within the survivor group. ROC curve analysis was applied to the PF ratio, OI, and OSI, reporting the AUC with its 95% CI, sensitivity, specificity, positive and negative predictive values, and the optimal cutoff derived by the Youden index. The AUCs were reported individually and, in view of the sample size, were not compared by a paired statistical test. No multivariable model was fitted, with the severity scores required for adjustment not having been recorded. The significance level was considered as p<0.05.

Ethical considerations

Ethical approval was obtained from the Institutional Review Board (IRB) of Bangladesh Medical University prior to the initiation of the study (approval number: BSMMU/2024/5688). All participants were receiving invasive mechanical ventilation and were sedated or otherwise incapable of providing consent at the time of enrolment. Written informed consent was therefore obtained in every case from the legally authorized representative, namely, the legal guardian or next of kin, in accordance with the protocol approved by the IRB. No patient was enrolled under a waiver of consent, and consent was not sought directly from any patient, since none was capable of giving it at enrolment. The confidentiality and anonymity of patient data were strictly maintained throughout the study. The study was conducted in accordance with the ethical principles outlined in the Declaration of Helsinki.

## Results

Of the 80 patients enrolled, 45 (56.3%) died in the ICU, and 35 (43.8%) survived to ICU discharge. All 80 contributed a complete set of day-1 measurements of the PF ratio, OI, and OSI. Table 2 presents the baseline sociodemographic characteristics of the study population. The participants were predominantly middle-aged, with a mean age of 47.58±20.29 years, and the majority (57, 71.2%) were aged ≤60 years, whereas 23 (28.8%) were older than 60 years. A greater proportion of the study population was male (53, 66.2%), whereas 27 (33.8%) were female. In terms of nutritional status, 36 (45%) participants had a normal BMI, followed by 25 (31.3%) who were overweight, 10 (12.4%) who were obese, and nine (11.3%) who were underweight.

**Table 2:** Baseline demographic characteristics of the study population (n=80) Data are expressed as frequencies (percentages) or mean±SD. BMI: body mass index

Variable	Category	n (%)
Age (years)	Mean±SD	47.58±20.29
	≤60 years	57 (71.2)
	>60 years	23 (28.8)
Sex	Male	53 (66.2)
	Female	27 (33.8)
BMI (kg/m²)	Underweight (<18.5)	9 (11.3)
	Normal (18.5-24.9)	36 (45)
	Overweight (25-29.9)	25 (31.3)
	Obese (≥30)	10 (12.4)

The comparison between survivors and non-survivors is shown in Table 3. There was no statistically significant difference in age between non-survivors (46.0±17.12 years) and survivors (48.6±24.08 years) (t(78)=0.43; p=0.667; Cohen's d=0.10; 95% CI: -0.34 to 0.54). Similarly, the sex distribution was comparable between the two groups, with males comprising 30 (66.7%) non-survivors and 23 (65.7%) survivors (χ²(1)=0.009; p=0.925; Cramér's V=0.011). BMI categories were also similarly distributed between the groups (χ²(3)=1.488; p=0.685; Cramér's V=0.136). Among the non-survivors, four (8.9%) were underweight, 15 (33.3%) were normal weight, 13 (28.9%) were overweight, and 13 (28.9%) were obese. Among the survivors, five (14.3%) were underweight, 14 (40%) were normal weight, eight (22.9%) were overweight, and eight (22.9%) were obese. With respect to comorbid conditions, diabetes mellitus was present in nine (20%) non-survivors and six (17.2%) survivors (χ²(1)=0.856; p=0.355; Cramér's V=0.103). Hypertension was observed in 10 (22.2%) non-survivors and six (17.2%) survivors (χ²(1)=1.472; p=0.225; Cramér's V=0.136). Ischemic heart disease was found in four (8.9%) non-survivors and three (8.6%) survivors, whereas chronic kidney disease was present in three (6.7%) non-survivors and three (8.6%) survivors (Fisher's exact test; p>0.99 for both).

**Table 3:** Comparison of demographic characteristics and comorbidities between non-survivors and survivors (n=80) The data are expressed as n (%) or the mean±SD. p<0.05 was considered statistically significant. BMI categories and comorbidities were compared via the chi-squared test, except for ischemic heart disease and chronic kidney disease, for which Fisher's exact test was used because of expected cell counts below 5. Test statistics are reported with their degrees of freedom (df); Cramér's V is given as the effect size for chi-squared tests and Cohen's d for the independent samples t-test. BMI: body mass index; χ²: chi-squared statistic

Variable	Non-survivors (n=45)	Survivors (n=35)	Test statistic (effect size)	P-value
Demographics				
Age, mean±SD (years)	46.0±17.12	48.6±24.08	t(78)=0.43 (d=0.10; 95% CI: -0.34 to 0.54)	0.667
Male sex, n (%)	30 (66.7)	23 (65.7)	χ²(1)=0.009 (V=0.011)	0.925
BMI (kg/m²), n (%)				
Underweight	4 (8.9)	5 (14.3)	χ²(3)=1.488 (V=0.136)	0.685
Normal weight	15 (33.3)	14 (40)		
Overweight	13 (28.9)	8 (22.9)		
Obese	13 (28.9)	8 (22.9)		
Comorbidities				
Diabetes mellitus	9 (20)	6 (17.2)	χ²(1)=0.856 (V=0.103)	0.355
Hypertension	10 (22.2)	6 (17.2)	χ²(1)=1.472 (V=0.136)	0.225
Ischemic heart disease	4 (8.9)	3 (8.6)	Fisher's exact test	>0.99
Chronic kidney disease	3 (6.7)	3 (8.6)	Fisher's exact test	>0.99

Table 4 shows the comparison of hemodynamic and ventilatory parameters between the two groups at ICU admission (day 1). Compared with survivors, non-survivors had significantly lower systolic blood pressure (102.68±25.12 mmHg vs. 120.36±31.73 mmHg; p=0.034) and diastolic blood pressure (56.64±15.92 mmHg vs. 71.96±21.82 mmHg; p=0.007). In contrast, non-survivors presented significantly higher heart rates (120.36±19.14 vs. 96.28±24.54 beats/min; p<0.001) and respiratory rates (30.96±7.68 vs. 22.00±6.87 breaths/min; p<0.001). With respect to ventilatory parameters, non-survivors required significantly greater PEEP (6.40±1.15 vs. 5.68±0.95 cmH₂O; p=0.020) and tidal volume (460.80±30.95 vs. 427.60±33.33 mL; p=0.001). These patients also had significantly lower PaO₂ values (53.68±19.12 vs. 72.44±21.15 mmHg; p=0.002) and SpO₂ values (77.58±17.53 vs. 91.53±6.03%; p<0.001), along with higher FiO₂ requirements (0.90±0.15 vs. 0.76±0.26; p=0.025). The mean airway pressure did not differ significantly between the groups (p=0.530). The difference in PEEP between the groups, although statistically significant, amounted to 0.72 cmH₂O, which is of limited clinical importance, and a similar caution applies to the difference in tidal volume.

**Table 4:** Comparison of hemodynamic and ventilator parameters between non-survivors and survivors on day 1 (n=80) The data are expressed as the mean±SD. *p<0.05 by independent t-test. SBP/DBP: systolic/diastolic blood pressure; PEEP: positive end-expiratory pressure; PaO₂: partial pressure of arterial oxygen; SpO₂: peripheral oxygen saturation; FiO₂: fraction of inspired oxygen

Parameter	Non-survivors (n=45) mean±SD	Survivors (n=35) mean±SD	P-value
Hemodynamics			
SBP (mmHg)	102.68±25.12	120.36±31.73	0.034*
DBP (mmHg)	56.64±15.92	71.96±21.82	0.007*
Heart rate (beats/min)	120.36±19.14	96.28±24.54	<0.001*
Respiratory rate (/min)	30.96±7.68	22.00±6.87	<0.001*
Ventilator and oxygenation			
PEEP (cmH₂O)	6.40±1.15	5.68±0.95	0.020*
Tidal volume (mL)	460.80±30.95	427.60±33.33	0.001*
PaO₂ (mmHg)	53.68±19.12	72.44±21.15	0.002*
SpO₂ (%)	77.58±17.53	91.53±6.03	<0.001*
FiO₂	0.90±0.15	0.76±0.26	0.025*
Mean airway pressure (cmH₂O)	13.18±3.54	12.53±3.69	0.530

A comparison of oxygenation indices is presented in Table 5. Compared with survivors, non-survivors had significantly greater OIs (43.71±28.48 vs. 16.18±12.36; t(78)=2.40; p=0.019; Cohen's d=0.54; 95% CI: 0.09 to 0.99) and OSIs (28.15±15.74 vs. 11.25±7.25; t(78)=2.29; p=0.025; Cohen's d=0.52; 95% CI: 0.07 to 0.97). Conversely, the PF ratio was significantly lower among non-survivors (62.98±27.94 vs. 115.03±71.71; t(78)=3.42; p=0.001; Cohen's d=0.77; 95% CI: 0.31 to 1.23). In terms of the Berlin ARDS classification, severe ARDS (PF ratio <100) was observed in 36 (80%) non-survivors and 22 (62.9%) survivors. Moderate ARDS was present in nine (20%) non-survivors and 11 (31.4%) survivors, whereas mild ARDS was observed only among survivors (two (5.7%)). However, the overall distribution of the Berlin ARDS severity categories did not differ significantly between the groups (χ²(2)=2.781; p=0.249; Cramér's V=0.186).

**Table 5:** Comparison of oxygenation severity indices and the Berlin ARDS classification between non-survivors and survivors on day 1 (n=80) The data are expressed as the mean±SD or n (%). *p<0.05 by independent t-test. The Berlin severity classification was based on the PF ratio and was compared via the chi-squared test. Test statistics are reported with their degrees of freedom (df); Cohen's d is given as the effect size for t-tests and Cramér's V for the chi-squared test. ARDS: acute respiratory distress syndrome; χ²: chi-squared statistic. OI: oxygenation index; OSI: oxygen saturation index; PF: partial pressure of arterial oxygen (PaO₂)/fraction of inspired oxygen (FiO₂)

Index	Non-survivors (n=45) mean±SD	Survivors (n=35) mean±SD	Test statistic (effect size)	P-value
OI	43.71±28.48	16.18±12.36	t(78)=2.40 (d=0.54; 95% CI: 0.09 to 0.99)	0.019*
OSI	28.15±15.74	11.25±7.25	t(78)=2.29 (d=0.52; 95% CI: 0.07 to 0.97)	0.025*
PF ratio	62.98±27.94	115.03±71.71	t(78)=3.42 (d= 0.77; 95% CI: 0.31 to 1.23)	0.001*
Berlin severity, n (%)				
Mild	0 (0)	2 (5.7)	χ²(2)=2.781 (V=0.186)	0.249
Moderate	9 (20)	11 (31.4)		
Severe	36 (80)	22 (62.9)		

Table 6 summarizes the temporal changes in oxygenation indices among survivors (n=35). The 45 patients who died contributed no day-3 or day-7 values because the indices are undefined after death, so this analysis is restricted to the 35 survivors and describes their trajectory rather than that of the whole cohort. Although the PF ratio increased from 115.03±71.71 on day 1 to 136.2±116.6 on day 7, the change was not statistically significant (p=0.109). Similarly, the OI and OSI gradually improved over time, but none of these changes reached statistical significance (p>0.05 for all comparisons).

**Table 6:** Serial changes in oxygenation indices over time in the survivor group (n=35) The data are expressed as the mean±SD. P-values were obtained via paired t-tests. OI: oxygenation index; OSI: oxygen saturation index; PF: partial pressure of arterial oxygen (PaO₂)/fraction of inspired oxygen (FiO₂); D: day

Index	Day 1 mean±SD	Day 3 mean±SD	Day 7 mean±SD	P-value	
				D1 vs. D3	D1 vs. D7
OI	16.18±12.36	15.2±10.3	12.2±10.0	0.061	0.231
OSI	11.25±7.25	11.0±4.8	10.7±5.6	0.861	0.777
PF ratio	115.03±71.71	130.3±66.8	136.2±116.6	0.411	0.109

The diagnostic performance of the oxygenation indices is presented in Table 7. The AUC was numerically highest for the OSI (0.879; 95% CI: 0.786-0.977; p<0.001), followed by the OI (0.867; 95% CI: 0.788-0.963; p<0.001) and the PF ratio (0.731; 95% CI: 0.612-0.881; p=0.003). At the optimal cutoffs, specificity was 91.4% for both the OSI and the OI but 60% for the PF ratio, and overall accuracy was 86.3%, 83.8%, and 70%, respectively. All 80 patients, comprising 45 deaths and 35 survivors, contributed to each ROC curve analysis. Because accuracy can refer to several different measures and is misleading when outcomes are imbalanced, the comparison between indices is framed in terms of the AUC rather than accuracy throughout this report. Two qualifications apply. The curves are correlated and were not compared by a paired test, so this ordering describes point estimates only, and the overlapping intervals for the OSI and OI indicate that these two cannot be distinguished at this sample size. The cutoffs were derived within the same dataset and are exploratory rather than established thresholds.

**Table 7:** ROC curve analysis and diagnostic performance of the OI, OSI, and PF ratio for mortality prediction Optimal cutoff values are determined by the Youden index. AUC: area under the curve; ROC: receiver operating characteristic; CI: confidence interval; OI: oxygenation index; OSI: oxygen saturation index; PF: partial pressure of arterial oxygen (PaO₂)/fraction of inspired oxygen (FiO₂)

Parameter	OI (cutoff ≥26.41)	OSI (cutoff ≥15.66)	PF ratio (cutoff ≤78.57)
ROC curve analysis			
AUC (95% CI)	0.867 (0.788-0.963)	0.879 (0.786-0.977)	0.731 (0.612-0.881)
P-value	<0.001	<0.001	0.003
Diagnostic performance			
Sensitivity (%)	76	80	80
Specificity (%)	91.4	91.4	60
Positive predictive value (%)	90.5	90.9	66.7
Negative predictive value (%)	79.3	82.1	75
Overall accuracy (%)	83.8	86.3	70

Figure 1 demonstrates the ROC curves of the OI, OSI, and PF ratio for mortality prediction. The OSI and OI curves showed better overall discrimination than the PF ratio, with the OSI demonstrating the greatest observed discriminatory ability among the three indices.

**Figure 1:**
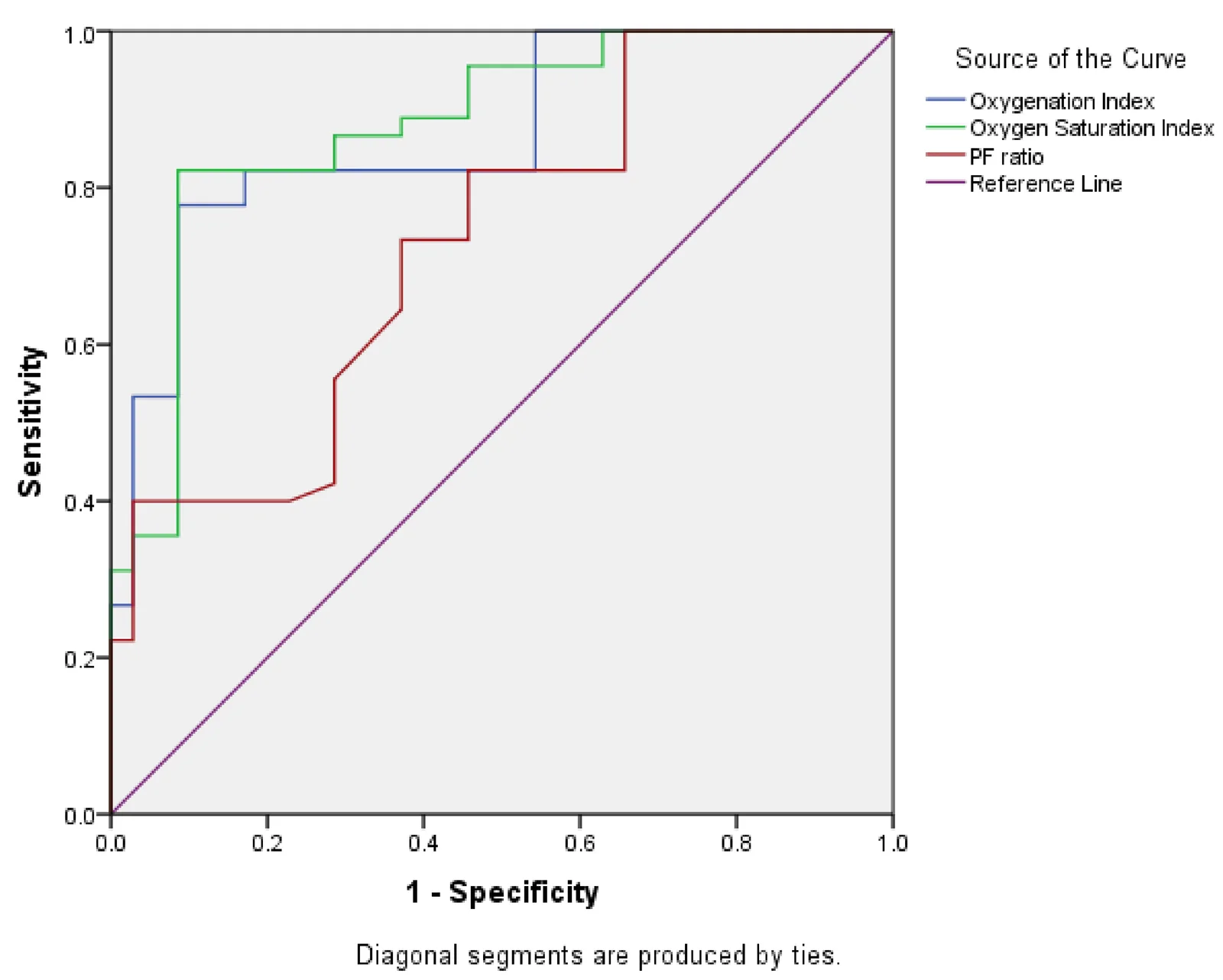
ROC curves of the OI, OSI, and PF ratio for mortality prediction ROC: receiver operating characteristic; OI: oxygenation index; OSI: oxygen saturation index; PF: partial pressure of arterial oxygen (PaO₂)/fraction of inspired oxygen (FiO₂)

## Discussion

In this study, we compared the PF ratio, OI, and OSI for mortality prediction in ARDS. The OI and OSI were higher and the PF ratio was lower among non-survivors, and both indices incorporating mean airway pressure showed numerically higher discrimination than the PF ratio, with the OSI recording the highest observed AUC. The wording used throughout is deliberately confined to discrimination and association. The analysis was univariable, and the correlated ROC curves were not formally compared, so these data show how well each index separates survivors from non-survivors, but not that one index is significantly better than another or that any carries prognostic information independent of overall illness severity.

Non-survivors also had a markedly worse profile at ICU admission, with lower systolic and diastolic pressures and higher heart and respiratory rates. This accords with evidence that hypotension and tachycardia at admission are associated with adverse outcomes in ARDS [21], a pattern usually reflecting systemic inflammation, hypoxia-driven myocardial impairment, increased right ventricular afterload, and the effects of positive-pressure ventilation [22]. They also required higher PEEP and tidal volumes with lower PaO₂ and SpO₂, suggesting more refractory hypoxemic failure, consistent with the cohort of Yu et al. in which greater oxygenation impairment and support requirements tracked ICU mortality [23]. Comorbidity distribution was comparable between groups, reinforcing acute severity as the dominant determinant of outcome in this population.

The physiological rationale for the higher discrimination of the OI and OSI lies in their inclusion of mean airway pressure. The PF ratio reflects gas exchange in isolation and varies with PEEP, tidal volume, and other settings irrespective of true lung injury severity [7], whereas the OI and OSI also capture the mechanical load imposed by ventilatory support. Our observations are consistent with this, although the design cannot confirm it. DesPrez et al. found that the OI outperformed the PF ratio for ICU and hospital mortality [11], Balzer et al. found that day-3 OI predicted 28-day mortality better than Berlin severity [24], and the OXIVA-CARDS investigators found that both indices predicted mortality in adults [25]. Two qualifications matter. Because mean airway pressure is clinician-set, the apparent advantage may partly reflect the intensity of support delivered rather than intrinsic lung injury, since patients on more aggressive support register higher values regardless of pathology. Moreover, most of the cited studies did not test the difference between indices statistically either, so the concordance described is one of observed point estimates across cohorts rather than accumulated evidence of superiority.

The OSI performed comparably to the OI while requiring no arterial sampling, being derived entirely from continuous pulse oximetry and ventilator-derived mean airway pressure. This is attractive where ABG access is intermittent, as in Bangladesh and other low- and middle-income countries [26]. Pulse oximetry-based indices are continuously available, inexpensive, and increasingly used for severity assessment in such settings [10], the OXIVA-CARDS investigators concluded that the OSI can assess hypoxemic failure without arterial access [25], and continuous monitoring avoids the vascular injury, hematoma, and infection risks of arterial puncture [10]. These advantages must nonetheless be weighed before the OSI is proposed as an alternative. The oxyhemoglobin dissociation curve is sigmoidal, so saturations above roughly 97% correspond to a wide range of PaO₂, and the index loses resolution where oxygenation is least impaired. Accuracy is further degraded by darker skin pigmentation, poor peripheral perfusion and shock, high-dose vasopressors, motion artefact, probe positioning, anemia, and dyshemoglobinemias, and occult hypoxemia has been described most often in darker-skinned patients. The OSI also inherits the treatment-intensity dependence described above, and our analysis does not establish that it adds information beyond the PF ratio once overall severity is considered. The OSI is therefore best regarded at present as a complement to rather than a replacement for ABG analysis, with attention to signal quality and periodic arterial confirmation, and it merits further evaluation as an adjunctive monitoring tool, particularly in these settings.

This study offers prospective evidence from a resource-limited ICU, with standardized collection and serial measurement. The design is single-center and observational, without long-term follow-up. The correlated AUCs were deliberately not subjected to a paired test, because a sample fixed by a proportion-estimation calculation cannot support one interpretably; the confidence intervals are reported instead, those for the OSI and the OI overlap substantially, and accordingly no superiority of any index over another is claimed. For the same reason of interpretability, and because Acute Physiology and Chronic Health Evaluation (APACHE) II and Sequential Organ Failure Assessment (SOFA) were not recorded prospectively, no multivariable model was fitted, so the analysis is univariable and independence from overall illness severity is neither established nor asserted. The study was powered to estimate a proportion, not to detect an AUC difference, so the ranking may be unstable. Cutoffs were internally derived and unvalidated, and discrimination alone does not establish clinical usefulness, with calibration and net benefit remaining unexamined. Serial data were missing through death, so the temporal analysis is survivor-restricted and descriptive. Berlin categories did not differ, unsurprisingly, since 58 of 80 patients (72.5%) had severe ARDS and that grading rests on the least discriminating index. Several continuous variables are skewed, so median, interquartile range, and non-parametric testing would be an alternative to the parametric approach retained here for comparability with the published literature on these indices; the group differences concerned are large, and their direction is not in doubt, so the choice of summary does not affect any conclusion drawn. Finally, the study compared only the statistical discrimination of the three indices and did not evaluate ABG availability, cost, turnaround time, or procedural complications, so the practical case for the OSI rests on discrimination alone and remains to be demonstrated.

## Conclusions

The present study showed that the OI and OSI were higher among non-survivors and discriminated ICU mortality numerically better than the PF ratio, with the OSI recording the highest observed AUC. Because the analysis was univariable, the correlated AUCs were not formally compared, and the cutoffs were derived within the same sample, these findings describe prognostic discrimination and association rather than independent prognostic value, and the thresholds reported are exploratory. These findings are hypothesis-generating rather than confirmatory and require external validation before indices incorporating ventilatory parameters can be said to add prognostic information beyond the PF ratio alone. Larger multicenter studies with paired comparison of the correlated ROC curves, multivariable adjustment for established severity scores, and independent validation of cutoffs are needed to confirm the relative prognostic performance of these indices.
